# Dynamic metabolic adaptation can promote species coexistence in competitive microbial communities

**DOI:** 10.1371/journal.pcbi.1007896

**Published:** 2020-05-07

**Authors:** Leonardo Pacciani-Mori, Andrea Giometto, Samir Suweis, Amos Maritan

**Affiliations:** 1 Department of Physics and Astronomy “Galileo Galilei”, University of Padua, Padua, Italy; 2 Department of Physics and Department of Molecular and Cellular Biology, Harvard University, Cambridge, Massachusetts, United States of America; University of Illinois at Urbana-Champaign, UNITED STATES

## Abstract

Microbes are capable of physiologically adapting to diverse environmental conditions by differentially varying the rates at which they uptake different nutrients. In particular, microbes can switch hierarchically between different energy sources, consuming first those that ensure the highest growth rate. Experimentally, this can result in biphasic growth curves called “diauxic shifts” that typically arise when microbes are grown in media containing several nutrients. Despite these observations are well known in microbiology and molecular biology, the mathematical models generally used to describe the population dynamics of microbial communities do not account for dynamic metabolic adaptation, thus implicitly assuming that microbes cannot switch dynamically from one resource to another. Here, we introduce dynamic metabolic adaptation in the framework of consumer-resource models, which are commonly used to describe competitive microbial communities, allowing each species to temporally change its preferred energy source to maximize its own relative fitness. We show that dynamic metabolic adaptation enables the community to self-organize, allowing several species to coexist even in the presence of few resources, and to respond optimally to a time-dependent environment, thus showing that dynamic metabolic adaptation could be an important mechanism for maintaining high levels of diversity even in environments with few energy sources. We show that introducing dynamic metabolic strategies in consumer-resource models is necessary for reproducing experimental growth curves of the baker’s yeast *Saccharomyces cerevisiae* growing in the presence of two carbon sources. Even though diauxic shifts emerge naturally from the model when two resources are qualitatively very different, the model predicts that the existence of such shifts is not a prerequisite for species coexistence in competitive communities.

## Author summary

Since a celebrated experiment by Monod in 1949, we know that microbes can adapt their metabolic strategies in response to their environment, thus uptaking different nutrients at different rates depending on their relative quality and availability. It is currently unclear whether this physiological plasticity of microbes contributes to maintaining the high degree of diversity found in natural microbial communities, even in the presence of very few resources. By introducing dynamic and optimal metabolic strategies in consumer-resource models, we show that we can correctly reproduce the experimental growth of *Saccharomyces cerevisiae* on two different resources and we show that dynamic metabolic strategies allow the community to self-organize and to ensure the coexistence of multiple species competing for few resources.

## Introduction

Biodiversity is one of the most fascinating aspects of nature: from the microscopic to the continental scale, complex communities composed of tens to thousands of species compete for resources and yet coexist. In particular, the survival of a species depends on the availability of resources in the environment, which is not static and can be altered by the presence of other species in the community. Furthermore, biodiversity is crucial for the functioning and maintenance of whole ecosystems, directly impacting their productivity, stability and many other properties [1]. It is thus critical to understand what are the mechanisms that can promote and maintain biodiversity within natural communities. To answer this question, the coexistence of several species in the same ecosystem can be investigated experimentally using controlled microbial communities and theoretically using models of community dynamics in the presence of multiple resources. Indeed, studies in the field of microbial ecology have shown that several species can coexist in the presence of few resources [2–6], and how this is possible is a long-standing open question [6–9] that recent theoretical studies are trying to answer [10–12].

Independent and apparently unrelated experiments of microbial batch growth have shown that microbes are capable of physiologically adapting to environments containing two or more resources [13–15]. Microbes can in fact physiologically adapt to different environmental conditions in various ways, by transforming and recycling nutrients and by varying the rates with which they uptake resources with time. Already in the early 1940s, Jacques Monod [13] observed that *Escherichia coli* and *Bacillus subtilis* grown in a culture medium containing two different sugars exhibit a biphasic growth curve, which he called “diauxie”. Instead of metabolizing these two nutrients simultaneously, bacteria consumed them sequentially using the most favorable one first (i.e., the one that conferred the highest growth rate) and once it had been depleted, following a lag phase, they resumed growth using the other sugar. Since then, diauxic growth has been the subject of thorough empirical study [16–19], via experiments that have generally involved the growth of one microbe on two resources, and the occurrence of diauxic shifts has been documented to occur widely across different microbial species [20–22]. Many models have been proposed to describe this phenomenon of “metabolic dynamic adaptation”, but all are focused on the specific gene regulation and expression mechanisms of a given species [15, 23], and are generally tailored to describe the growth of such a species on a specific set of resources [24–26]. The general effects of using dynamic strategies on the maintenance of biodiversity in microbial communities, therefore, have not yet been investigated from the ecological dynamics perspective, with the exception of very few studies that have analyzed similar effects on different types of system: Valdovinos et al. [27], for example, investigated the consequences of adaptive foraging in plant-pollinator systems and found out that this effect increases species persistence and diversity.

In principle, the ability of microbial species to vary the rates at which they consume different nutrients might allow them to diversify the consumption of different resources in response to both the presence of other species and to the abundance and quality of the nutrients available, and this in turn might allow them to persist in the community. To understand the general implications of dynamic metabolic strategies for the maintenance of biodiversity, one needs to abstract from the specific molecular mechanisms that regulate the preferential consumption of different resources by any given species. What is needed, instead, is a general, phenomenological framework capable of describing these phenomena in a unified way as emergent properties of complex systems of agents that interact with each other and with the environment, rather than through *ad hoc* tailored biological and/or molecular mechanisms. Recently there has been a growing effort to develop such a framework, with particular focus on the conditions leading to species coexistence [10, 11, 28–32]. The models devised in this direction typically build on MacArthur’s consumer-resource framework [33, 34], describing the competition of species for a common pool of resources, but neglecting dynamic metabolic adaptation. In fact, despite the aforementioned evidence for dynamic metabolic adaptation from studies of microbial metabolism, these models implicitly but systematically assume that the metabolic strategies (defined here as the maximum resource uptake rates) of microbial species do not change with time, and assume that a species’ consumption rate of a given resource depends solely on the concentration of the latter, and not on the presence of other species, nor on the concentration of other nutrients. There are only a few examples in the literature where metabolic strategies in microbial communities are allowed to change to a certain degree, but these studies were neither focused on deriving conditions for species coexistence, especially as a consequence of having dynamic metabolic strategies, nor did they take into account a continuous temporal dynamics for metabolic strategies. Goyal et al. [12], for example, have recently developed a conceptual model of microbial communities showing that such systems can have multiple stable states, that they can restructure themselves after external perturbations, and that complementarity in nutrient preferences allows multiple species to coexist; in this model, species can switch instantaneously between different energy sources, but the model does not explicitly describe population dynamics. Marsland et al. [35], on the other hand, have considered models where resource uptake rates can be regulated so that species use the resource that is currently the most abundant, and species can excrete secondary metabolites into the environment.

In this work we allow metabolic strategies to depend on time within a consumer-resource model. The temporal dynamics of such metabolic strategies is set to maximize the relative fitness of each species. We show that this approach is capable of quantitatively reproducing experimentally-measured growth curves of *S. cerevisiae* consuming multiple resources, in contrast to a consumer-resource model with fixed strategies. When considering a community composed of multiple species consuming multiple resources, our model suggests that dynamic metabolic adaptation plays a major role in maintaining species diversity, especially when few common resources are available. Furthermore, if the environmental conditions of the system are variable over time, or if some of the available resources degrade rapidly, our adaptive framework is capable of maintaining the coexistence of several species on few resources, while the classical MacArthur’s consumer-resource model with fixed metabolic strategies would predict the extinction of most species. Our work therefore proposes a unifying theoretical framework capable of reproducing both the existence of diauxic shifts and the coexistence of a large number of species competing for a limited number of resources in various realistic ecological settings, thus suggesting that dynamic metabolic adaptation can play an important role in maintaining high levels of biodiversity in microbial communities.

When using consumer-resource models it is of paramount importance to identify what are the ‘‘resources’’. In fact, several properties of this type of models, particularly those relative to the maintenance of species diversity, depend crucially on identifying the growth-limiting resources. In this work we consider only substitutable resources, i.e. we identify as resources those substances present in the environment that can be used interchangeably for microbial growth (e.g. different sugars as carbon sources). Finally, we point out that since our framework is based on consumer-resource ecological models, the term ‘‘adaptation’’ is used here in the system dynamics sense (i.e., indicating changes in the metabolic state of a species aimed at maximizing its growth rate over ecological time scales) and should *not* be intended in the evolutionary sense (i.e., the process by which species become better adapted to their environment via mutation, genetic drift and selection over evolutionary times).

## Results

### The MacArthur’s consumer-resource model

In the classical formulation of MacArthur’s consumer-resource model, a community of *m* species competes for *p* resources according to the following equations:

$$\begin{array}{c} {\dot{n}}_{\sigma }=n_{\sigma }(\sum_{i=1}^{p}v_{i}\alpha_{\sigma i}r_{i}\left(c_{i}\right)-\delta_{\sigma })\;\;, \end{array}$$ (1)

$$\begin{array}{c} {\dot{c}}_{i}=s_{i}-\sum_{\sigma =1}^{m}n_{\sigma }\alpha_{\sigma i}r_{i}\left(c_{i}\right)-\mu_{i}c_{i}\;\;, \end{array}$$ (2)

where *n*_*σ*_(*t*) describes the population density of species *σ*, *c*_*i*_(*t*) is the concentration of resource *i* and *δ*_*σ*_ is the death rate of species *σ*. The quantity *r*_*i*_(*c*_*i*_) is a function accounting for the fact that the dependence of a species’ growth rate on a given resource concentration saturates as *c*_*i*_ is increased. Without loss of generality, we assume that *r*_*i*_(*c*_*i*_) has the form of a Monod function [13], i.e. *r*_*i*_(*c*_*i*_) = *c*_*i*_/(*K*_*i*_ + *c*_*i*_) with *K*_*i*_ > 0 (*K*_*i*_ is the half-saturation constant), and so *r*_*i*_(*c*_*i*_) < 1 ∀ *c*_*i*_ > 0. The quantities *α*_*σi*_ ≥ 0 are the metabolic strategies, and each one of them can be interpreted as the maximum rate at which species *σ* uptakes resource *i*. The parameter *v*_*i*_ is often called “resource value” and is related to the resource-to-biomass conversion efficiency: the larger *v*_*i*_, the larger the population growth rate that is achieved for unit resource quantity, and thus the more “favorable” resource *i* is. The parameter *s*_*i*_ is a constant nutrient supply rate, and the sum in (2) represents the action of all consumers on resource *i*. Such an action depends of course on the metabolic strategies *α*_*σi*_. Finally, *μ*_*i*_ ≥ 0 is the degradation rate of resource *i*.

### Introducing dynamic metabolic adaptation

Our introduction of dynamic metabolic strategies in the consumer-resource framework starts from the requirement that each metabolic strategy ${\vec{\alpha }}_{\sigma }={\left(\alpha_{\sigma 1},\ldots ,\alpha_{\sigma p}\right)}^{T}$ changes in time to maximize the relative fitness of species *σ*, measured [36, 37] as the growth rate $g_{\sigma }=\sum_{i=1}^{p}v_{i}\alpha_{\sigma i}r_{i}\left(c_{i}\right)-\delta_{\sigma }$. This can be achieved by requiring that metabolic strategies follow a simple gradient ascent equation:

$$\begin{array}{c} {\dot{\alpha }}_{\sigma i}\propto \frac{\partial g_{\sigma }}{\partial \alpha_{\sigma i}}\;\;. \end{array}$$ (3)

Notice that introducing adaptive metabolic strategies in the MacArthur’s consumer-resource model reduces the number of independent parameters, given that the *m* ⋅ *p* metabolic strategies become dynamical variables.

Eq (3) is missing an important biological constraint, which is related to intrinsic limitations to any species’ resource uptake and metabolic rates: by necessity, microbes have limited amounts of energy that they can use to produce the metabolites necessary for resource uptake, so we must introduce such a constraint in (3). To do so, we require that each species has a maximum total resource uptake rate $E_{\sigma }^{*}\geq 0$ that it can achieve, i.e. $\sum_{i=1}^{p}\alpha_{\sigma i}\left(t\right)≔E_{\sigma }\left(t\right)\leq E_{\sigma }^{*}$. The choice of imposing a soft constraint in the form of an inequality is not arbitrary, as it is rooted in the experimental evidence that microbes cannot devote an unbounded amount of energy to metabolizing nutrients. Experiments [38] have shown, in fact, that introducing a constraint for metabolic fluxes in the form of an upper bound (perfectly analogous to the one we adopted in this work, see Eq. (4) in [38]) allows one to improve the agreement between Flux Balance Analysis modeling and experimental data on *E. coli* growth on different substrates.

The constraint on the species’ maximum total resource uptake rates introduces a trade-off between the use of different resources. In S1 Text we present a geometrical interpretation of the maximization problem given by (3), i.e. ${\dot{\vec{\alpha }}}_{\sigma }\propto {\vec{\nabla }}_{{\vec{\alpha }}_{\sigma }}g_{\sigma }$ where ${\vec{\nabla }}_{{\vec{\alpha }}_{\sigma }}$ is the gradient with respect to the components of ${\vec{\alpha }}_{\sigma }$. In particular, since we want ${\vec{\alpha }}_{\sigma }$ to change so that the constraint $\varphi \left({\vec{\alpha }}_{\sigma }\left(t\right)\right)≔\sum_{i=1}^{p}\alpha_{\sigma i}\left(t\right)/E_{\sigma }^{*}-1\leq 0$ is satisfied, we remove from ${\vec{\nabla }}_{{\vec{\alpha }}_{\sigma }}g_{\sigma }$ the component parallel to ${\vec{\nabla }}_{{\vec{\alpha }}_{\sigma }}\varphi \left({\vec{\alpha }}_{\sigma }\left(t\right)\right)$ as soon as $\varphi ({\vec{\alpha }}_{\sigma }\left(t\right))=0$. Furthermore, we prevent the metabolic strategies from becoming negative. Eventually, the final equation for the metabolic strategies’ dynamics is given by (see S1 Text for the full derivation):

$$\begin{array}{c} {\dot{\alpha }}_{\sigma i}=\alpha_{\sigma i}\lambda_{\sigma }[v_{i}r_{i}-\frac{\Theta (\varphi ({\vec{\alpha }}_{\sigma }))}{\sum_{k=1}^{p}\alpha_{\sigma k}}\sum_{j=1}^{p}v_{j}r_{j}\alpha_{\sigma j}], \end{array}$$ (4)

where we have written *r*_*i*_ = *r*_*i*_(*c*_*i*_), Θ is Heaviside’s step function (i.e. Θ(*x*) = 1 when *x* ≥ 0 and Θ(*x*) = 0 otherwise) and λ_*σ*_ is the ‘‘learning rate’’ of species *σ*. Here, we assume that all the degradation rates *μ*_*i*_ are null, but we discuss a more general case below. Table 1 summarizes the parameters used in the model. See S1 Text for the detailed dimensional analysis of the parameters.

**Table 1:** Parameters used in our model, with their definition and units (see S1 Text for the detailed dimensional analysis of the model).

Parameter	Definition	Units
*n*_*σ*_	Population density of species *σ*	cell/mL
*δ*_*σ*_	Death rate of species *σ*	1/h
λ_*σ*_	Learning rate	g of resource /(cell ⋅ h)
$E_{\sigma }^{*}$	Total uptake rate of species *σ*	g of resource /(cell ⋅ h)
*α*_*σi*_	Metabolic strategy	g of resource /(cell ⋅ h)
*v*_*i*_	Value of resource *i*	cell/(g of resource)
*c*_*i*_	Density of resource *i*	g of resource/mL
*K*_*i*_	Half-saturation constant of resource *i*	g of resource/mL
*s*_*i*_	Supply rate of resource *i*	g of resource/(mL ⋅ h)
*μ*_*i*_	Degradation rate of resource *i*	1/h

### Diauxic shifts

**Fig 1:**
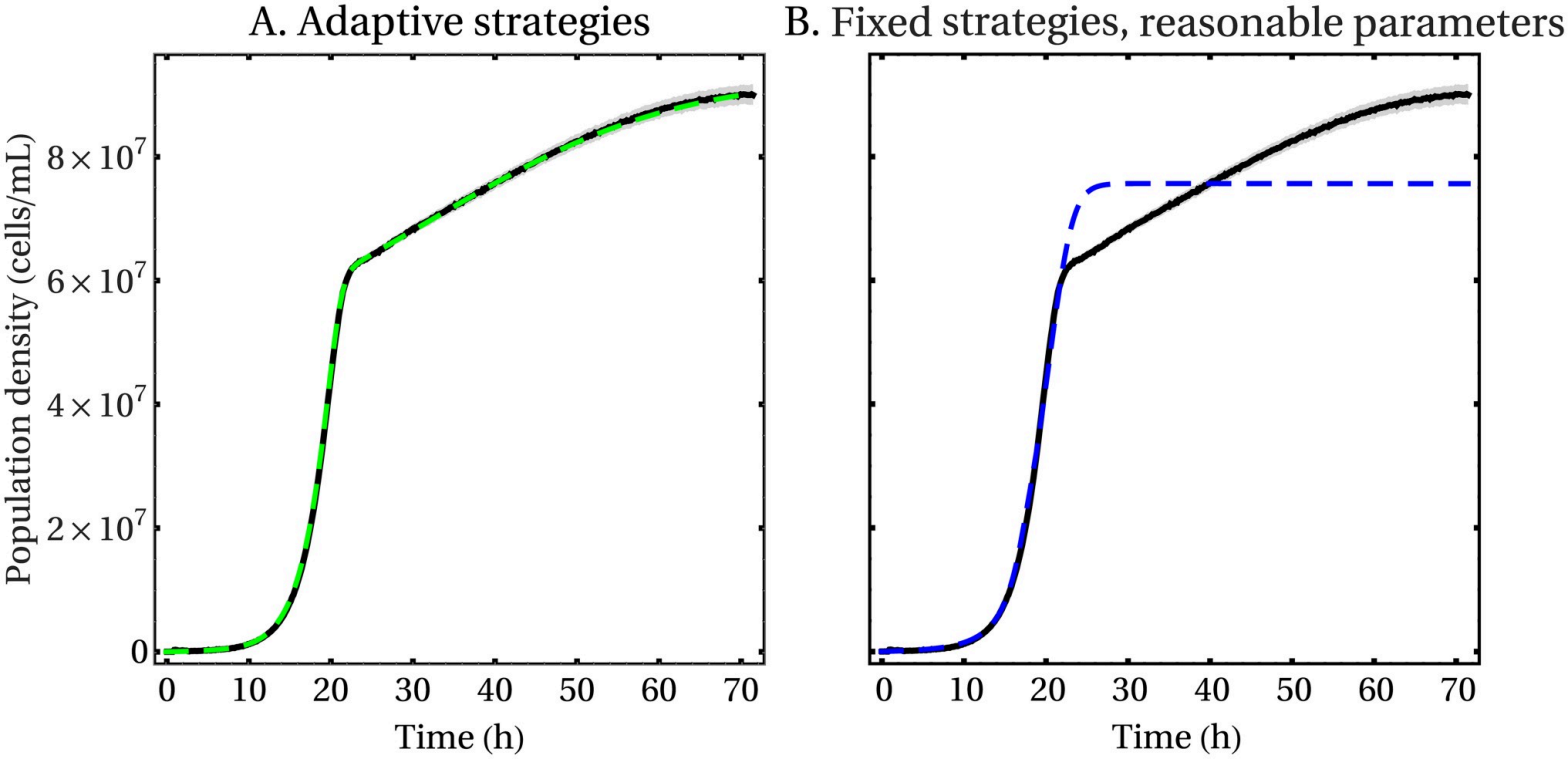
Comparison between the best fits of MacArthur’s consumer-resource model (dashed lines) and experimental measures of the growth of *S. cerevisiae* on galactose as the primary carbon source and ethanol as a byproduct of fermentation, in the case of adaptive (A) and fixed (B) metabolic strategies. Shown are the mean (black lines) and the standard error (gray bands) across *n* = 8 replicate populations. In (A) the model is not only capable to reproduce very well the experimental data, but the best fit returns parameters whose values are biologically reasonable when contrasted with experimentally-measured ones found in the literature (see Table A in S1 Text). On the other hand, the fit in (B) cannot reproduce a diauxic behavior when the parameters are constrained to vary within a few orders of magnitude away from biologically reasonable values (see Table A in S1 Text). See S1 Text for details on how the fits were performed and the resulting values of the best fit parameters.

If we introduce dynamic metabolic adaptation in a consumer-resource model so that each species changes its metabolic strategies to maximize its own growth rate, the new model is capable not only of reproducing qualitatively the growth dynamics of diauxic shifts, but to do so in quantitative agreement with experimental observations. To show this, we measured growth curves of the baker’s yeast, *S. cerevisiae*, grown in the presence of galactose as the primary carbon source. In these growth conditions, *S. cerevisiae* partially respires and partially ferments the sugar. As a byproduct of fermentation, yeast cells release ethanol in the growth medium, which can then be respired by the cells once the concentration of galactose in the medium is reduced. To model the growth of *S. cerevisiae* in these conditions, we modified the equations to account for the fact that the second resource, ethanol, is produced by the yeast cells themselves, while the first one, galactose, is consumed. We have then fitted the model to the data using a Markov Chain Monte Carlo (MCMC) algorithm [39] (see Methods). In Fig 1A, we show that our adaptive consumer-resource model can fit the experimental data with parameters that are compatible with values found in the literature (see Table A in S1 Text). When fitting the “classic” MacArthur’s consumer-resource model with fixed metabolic strategies, on the other hand, the same MCMC fitting algorithm returns two possible different outcomes, depending on the ranges that the parameters are allowed to explore in the Markov chain dynamics. When the parameters are constrained to vary within a few orders of magnitude from experimentally-measured values found in the literature (Table A in S1 Text), the fixed-strategies model is incapable of reproducing even a diauxic behavior (Fig 1B). When the parameters are subject to looser constraints on the value they can take, instead, the model can reproduce the data (Figure A in S1 Text), although not as well as the adaptive-strategies model, but some of the best fit parameters have biologically unreasonable values (see Table A in S1 Text). The Akaike Information Criterion, used to compare the relative quality of the two models discounting the number of parameters, selects unambiguously the model with adaptive strategies as the best fitting one when comparing it to either fits of the fixed-strategies model (see Methods).

### Species coexistence

The MacArthur’s consumer-resource model also makes predictions for the coexistence of *m* species on *p* shared resources and reproduces the so-called “Competitive Exclusion Principle” [40] (CEP), a theoretical argument that has sparked a lively debate in the ecological community [41–45]. According to the CEP, the maximum number of species that can stably coexist is equal to *p*. In nature, however, there are many situations in which the CEP appears to be violated: the most famous example of such violation is the “Paradox of the Plankton” [7], whereby a very high number of phytoplankton species is observed to coexist in the presence of a limited set of resources [46]. Many different mechanisms have been proposed to explain the violation of the CEP, ranging from non-equilibrium phenomena [7] to the existence of additional limiting factors like the presence of predators [47], cross-feeding relationships [6], toxin production [48], and complex or higher-order interactions [49, 50]; see [8] and [9] for comprehensive reviews.

**Fig 2:**
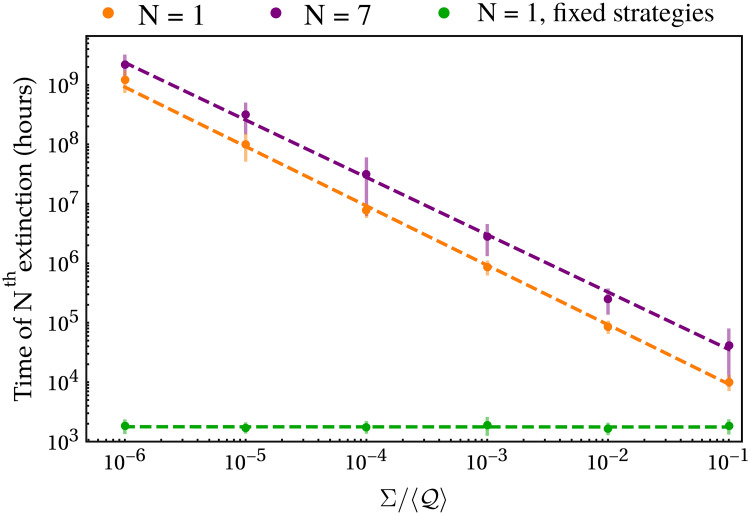
Time of first (orange) and seventh (purple) extinction in the consumer-resource model with adaptive metabolic strategies and with $E_{\sigma }^{*}$ drawn independently of *δ*_*σ*_. We used *m* = 10, *p* = 3 and $E_{\sigma }^{*}=Q_{\sigma }\delta_{\sigma }$ with $Q_{\sigma }$ drawn from a normal distribution with mean 〈Q〉 and standard deviation Σ; see S1 Text for more details on the parameters used. The extinction times were computed as the instants at which the densities of the species fell below 1 cell/mL. Both axes are in logarithmic scale, the error bars represent one standard deviation across 50 iterations of the model and the dashed lines are the best power-law fits. The behavior of the extinction times suggests that if Σ = 0 then all species could coexist indefinitely. Indeed, it is possible to show analytically that when $Q_{\sigma }=Q\;\;\forall \sigma$, all species coexist at the stationary state of the system (see S1 Text for details). The green points show, for comparison, the time of first extinction for a system with the same parameters but where metabolic strategies are fixed. As we can see, even when each species has its own CTR, $Q_{\sigma }$, using dynamic metabolic strategies increases by several orders of magnitude the length of the time interval over which species manage to coexist. The results shown do not change noticeably if the initial conditions on the populations are increased, even if by some orders of magnitude.

Considering now our model in the general case of *m* species and *p* resources, if the total maximum resource uptake rates $E_{\sigma }^{*}$ are completely uncorrelated to the death rates *δ*_*σ*_ (e.g. if $E_{\sigma }^{*}=Q_{\sigma }\delta_{\sigma }$, with $Q_{\sigma }>0$ drawn randomly from a given distribution with average 〈Q〉 and standard deviation Σ) we observe extinctions, i.e. in the infinite time limit, we cannot have more than *p* coexisting species (see S1 Text). We focus on the idealized case of infinite temporal coexistence to avoid the introduction of too many finite temporal scales, as would be the case when considering the inevitable perturbations experienced by communities that jeopardize their coexistence. In Fig 2 we show how the times of first and seventh extinction change as we vary the coefficient of variation Σ/〈Q〉 of the normal distribution from which we draw the $Q_{\sigma }$ in a system of *m* = 10 species and *p* = 3 resources. As shown, these extinction times increase sensibly as Σ/〈Q〉 is reduced. In other words, for $Q_{\sigma }$ more and more peaked around their mean value, the species present in the system can coexist for increasingly longer times. As we can see, the extinction times exhibit a power law-like behavior as a function of the coefficient of variation Σ/〈Q〉. In particular, we find that the times to extinction of the first *m* − *p* species scale approximately as ${\left(\Sigma /\langle Q\rangle \right)}^{-1}$. This observation suggests that adaptive strategies promote species biodiversity for finite time scales and that coexistence for an *infinite* time interval could be possible if the ratio between the maximum resource uptake rate $E_{\sigma }^{*}$ and the death rate of each species *δ*_*σ*_, which we call the Characteristic Timescale Ratio (CTR), does not depend on the species’ identities. In mathematical terms, if $E_{\sigma }^{*}/\delta_{\sigma }=Q_{\sigma }=Q\;\;\forall \sigma$, then *m* > *p* species can coexist. This requirement is compatible with the experimental observations that led to the formulation of the metabolic theory of ecology [51] (see S1 Text for a detailed mathematical justification of this statement), according to which these two rates ($E_{\sigma }^{*}$ and *δ*_*σ*_) depend only on the characteristic mass of a species. It is indeed possible to show analytically that our model can strictly violate the CEP if the CTR Q does not depend on the species’ identities (see S1 Text for details on the proof). In this latter case, since a single time scale characterizes each species, we set λ_*σ*_ = *dδ*_*σ*_, where *d* > 0 regulates the speed of adaptation.

For comparison, in Fig 2 we also show the times of first extinction for the same system with the same parameter distributions but where metabolic strategies are fixed. As we can see, these extinction times are all approximately equal independently of Σ/〈Q〉, and are orders of magnitude smaller than the ones obtained with dynamic metabolic strategies. It is therefore clear that even when each species has its own CTR, $Q_{\sigma }$, using dynamic metabolic strategies increases by several orders of magnitude the length of the time interval over which species coexist.

**Fig 3:**
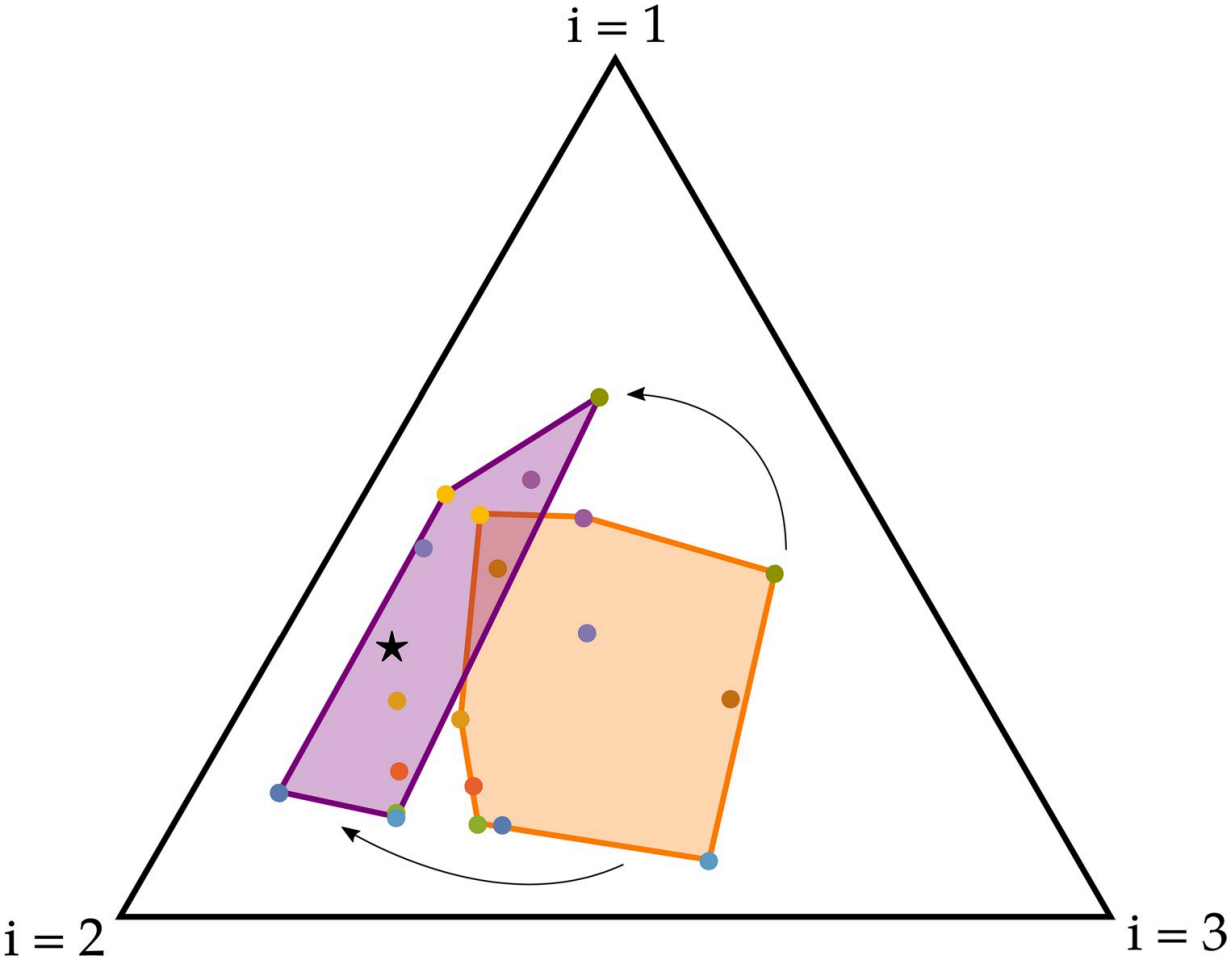
Comparison between the initial (orange) and final (purple) convex hull of the rescaled metabolic strategies (colored dots) when they are allowed to adapt according to (4). These results have been obtained for a system with *m* = 10 species and *p* = 3 resources using the graphical representation method introduced by Posfai et al. [10] and using a common value of the CTR Q for all species. In particular, in this case this method prescribes that the rescaled metabolic strategies and nutrient supply rate vector (black star) all lie on a 2-dimensional simplex (i.e. the triangle in the figure), where each vertex corresponds to one of the resources; for details on the parameters used, and for the plots of the temporal dynamics of the population densities and metabolic strategies, see Figure D in S1 Text. In the final state, the ${\vec{\hat{\alpha }}}_{\sigma }$ have incorporated $\vec{\hat{s}}$ in their convex hull.

MacArthur’s consumer-resource model with fixed *α*_*σi*_ has been shown to violate the CEP only if $\sum_{i=1}^{p}\alpha_{\sigma i}=E$ (where *E* > 0 is a constant independent of *σ*, i.e. the maximum resource uptake rate is the same for all species), if *δ*_*σ*_ = *δ*∀*σ* (where *δ* > 0 is a constant independent of *σ*, i.e. the death rate is the same for all species) and if $\vec{s}$, the vector whose components are the nutrient supply rates *s*_*i*_, belongs to the convex hull of the metabolic strategies ${\vec{\alpha }}_{\sigma }$ [10]. In general, any looser constraint (including $\sum_{i=1}^{p}\alpha_{\sigma i}\leq E_{\sigma }$ with arbitrary *E*_*σ*_) will lead to the extinction of at least *m* − *p* species, i.e. the system will obey the CEP; in this sense the system allows coexistence only when fine-tuned, a situation that is unlikely to be true for all natural communities. However, if we now use (4) for the dynamics of *α*_*σi*_, it is possible to show analytically that the system gains additional degrees of freedom which make it possible to find steady states where an arbitrary number of species can coexist, even when the initial conditions are not favorable. More specifically, if we denote by $\vec{\hat{s}}$ and ${\vec{\hat{\alpha }}}_{\sigma }$ some appropriately rescaled versions of the nutrient supply rate vector $\vec{s}$ and the metabolic strategies ${\vec{\alpha }}_{\sigma }$ (see S1 Text for more information), the system reaches a steady state where all species coexist even when $\vec{\hat{s}}$ initially does not belong to the convex hull of ${\vec{\hat{\alpha }}}_{\sigma }$. In Fig 3, we show the initial and final states of a temporal integration of the model: even though $\vec{\hat{s}}$ was initially outside the convex hull of ${\vec{\hat{\alpha }}}_{\sigma }$, the metabolic strategies changed to bring $\vec{\hat{s}}$ within the convex hull and thus allowed coexistence. Therefore, the community modeled by Eqs (1–4) is capable to *self-organize*. Notice that if we used fixed metabolic strategies in this case, almost all species would go extinct and the CEP would hold (see Figure D in S1 Text).

#### Minimization of energy waste

An independent prediction of our model is that if one of the available resources, e.g. resource *j*, is too energetically unfavorable, then dynamic metabolic adaptation will bring all the *j*-th components of the metabolic strategies to zero, i.e. species will stop using that resource. By what measure resource *i* is unfavorable is quantified by 1/*v*_*i*_. When the metabolic strategies are not allowed to adapt, it is possible to prove that a nontrivial stationary state (i.e. one where the CEP is violated) is possible only if $1/v_{i}<Q\;\;\forall i$; this means that if even just one of the resources is unfavorable, i.e. $1/v_{j}>Q$ for one *j*, then there will be extinctions and in the end the CEP will hold (see S1 Text and Figure E in S1 Text for more details). However, when we allow the strategies to adapt following (4), the system reaches a non-trivial stationary state even if there is one (or possibly more) resource *j* for which $1/v_{j}>Q$. In this case, in fact, resource *j* becomes too unfavorable, and it is possible to show that the system decouples from it, i.e. the *j*-th component of *all* the metabolic strategies becomes null (see Figure E in S1 Text). Something analogous happens also when degradation rates are present, i.e. *μ*_*i*_ > 0 in (2): in this case, at stationarity, the convex hull of the rescaled metabolic strategies will include the vector with components ${\tilde{s}}_{i}≔v_{i}\left(s_{i}-\mu_{i}c_{i}^{*}\right)/\sum_{j=1}^{p}v_{j}\left(s_{j}-\mu_{j}c_{j}^{*}\right)$ with $c_{i}^{*}$ the stationary value of *c*_*i*_(*t*) (see S1 Text), and if one of the *μ*_*i*_ is sufficiently large, this vector will lie on one of the sides of the (*p* − 1)-dimensional simplex where our system can be represented. In other words, we find that if the degradation rate *μ*_*j*_ of resource *j* becomes too large, then again all the *j*-th components of the metabolic strategies will become null (see Figures S5 and S7). On the other hand, if we introduce the resource degradation rates in MacArthur’s consumer-resource model with fixed metabolic strategies, extinctions will occur and the CEP will hold (see Figure F in S1 Text) for any choice of *E*_*σ*_. Therefore, species in our model minimize the energy they use to metabolize resources that are unfavorable or volatile, and they invest their energy budget on the more convenient ones.

#### Variable environmental conditions

**Fig 4:**
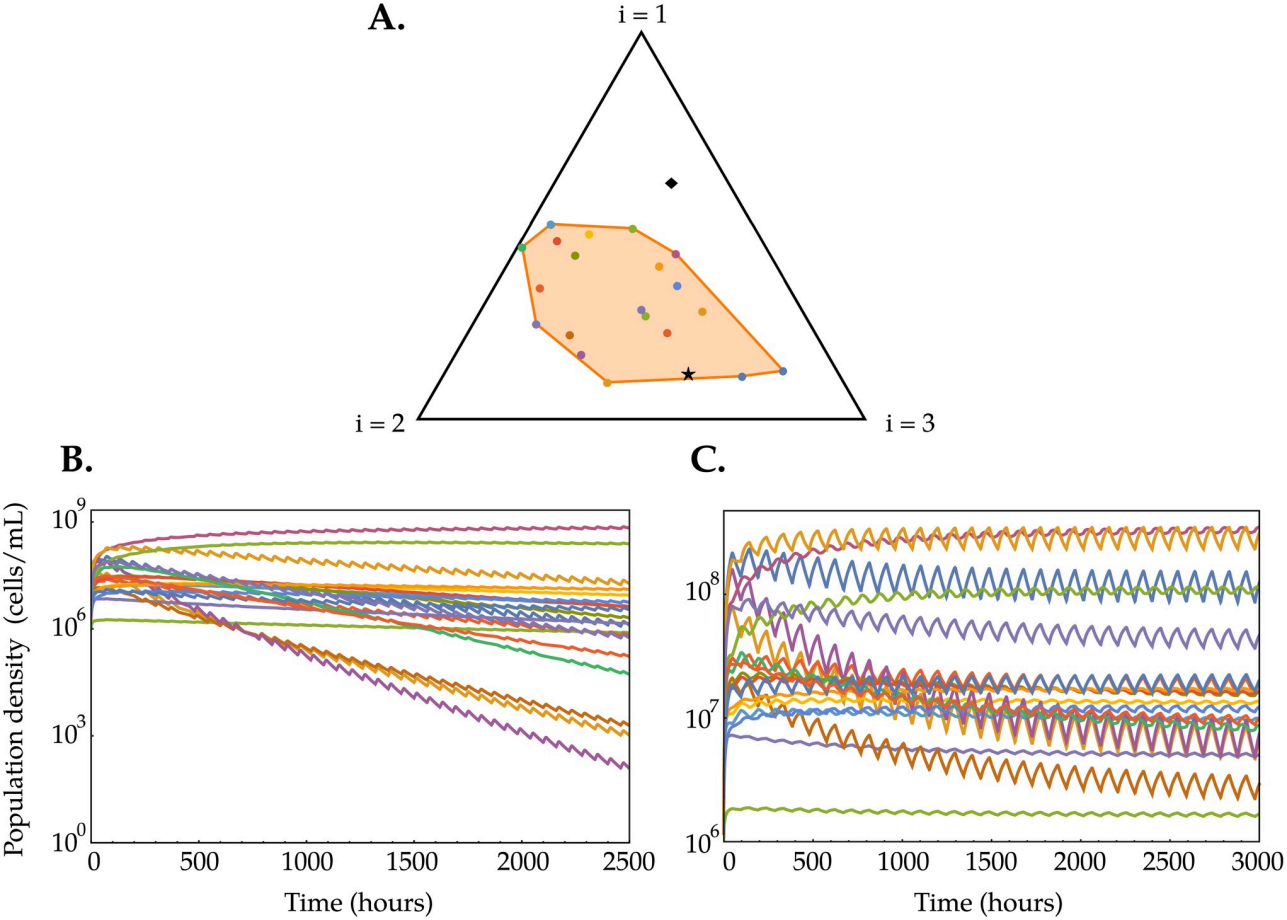
Comparison between the temporal dynamics of species’ population densities (each color represents a different species) in the consumer-resource models with fixed metabolic strategies, when the resource supply rate vector $\vec{s}$ varies with time. Here, we simulated a system with *m* = 20 species, *p* = 3 resources, and with the nutrient supply rate vector switching at regular intervals between the two values shown (black star and diamond) in (A). Specifically, in (B) we made $\vec{s}\left(t\right)$ alternate periodically between ${\vec{s}}_{\text{in}}$ for *τ*_in_ = 12 h and ${\vec{s}}_{\text{out}}$ for *τ*_out_ = 48 h, with ${\vec{s}}_{\text{in}}$ chosen within the convex hull of the initial rescaled metabolic strategies and ${\vec{s}}_{\text{out}}$ chosen outside of it (see Figure G in S1 Text for more information on the parameters used). Panel (C) shows the same quantities, with *τ*_in_ = *τ*_out_ = 48 h. See Figure G in S1 Text for the dynamics of the species’ populations in the consumer-resource model with adaptive metabolic strategies.

Having adaptive metabolic strategies also allows the system to better respond to variable environmental conditions, i.e. when $\vec{s}$ is a function of time $\vec{s}\left(t\right)$. Let us consider a scenario where the nutrient supply rates change periodically; this can be implemented by shifting $\vec{s}$ between two different values at regular time intervals: one inside the convex hull of the initial (rescaled) metabolic strategies and one outside of it. We found that when the metabolic strategies ${\vec{\alpha }}_{\sigma }$ are allowed to adapt, the species’ populations oscillate between two values and manage to coexist, while when the metabolic strategies are fixed in time, some species go extinct due to the perturbations and the CEP is recovered, unless $\vec{s}\left(t\right)$ spends enough time inside the convex hull of the metabolic strategies—see Fig 4. Also in the case of environmental conditions that vary with time, we find that when we introduce resource degradation rates that are sufficiently large, all the *i*-th components of the metabolic strategies vanish (see Figure H in S1 Text). Therefore, adaptive metabolic strategies allow species in the community to self-organize and efficiently deal with variable environmental conditions and a mix of (energetically) favorable and unfavorable resources, features characterizing natural ecosystems.

#### Adaptation velocity

**Fig 5:**
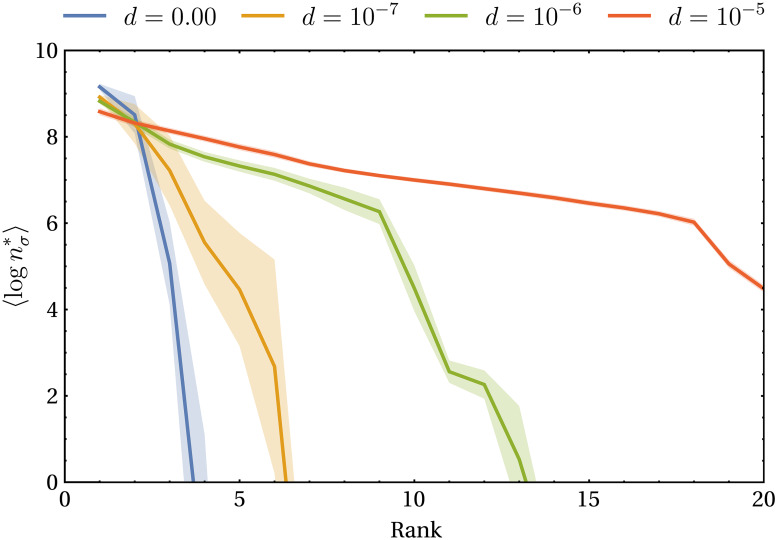
Rank distribution of the (decimal) logarithm of the stationary population densities $n_{\sigma }^{*}$ for different values of the adaptation velocity *d* (see Figure J in S1 Text for more information on the parameters used). The lines represent the average value over 100 iterations, while the opaque bands outline the standard error of the mean. For *d* = 0 (blue line) the rank distribution is very steep and only the first few species have a population density over 1 cell/mL (corresponding to $\text{log}\;n_{\sigma }^{*}=0$), while as *d* increases the distribution becomes more even. Setting $\text{log}\;n_{\sigma }^{*}=0$ as the extinction threshold, approximately two thirds of the species in the system go extinct with *d* = 10^−7^ (yellow line), while all of them survive with *d* = 10^−5^.

A physically relevant parameter characterizing the capacity of a species to adapt to a new environment is *d*, which regulates the velocity of dynamic metabolic adaptation for the metabolic strategies (see (3) with λ_*σ*_ = *dδ*_*σ*_, by which *d* has units of g of resource/cell). Increasing the value of *d* leads to metabolic strategies that adapt more rapidly, and as a consequence species’ growth rates will be optimized for longer periods of time. Thus, in a community in which the CTR is the same for all species, stationary population densities will be higher for larger values of *d*. When *d* tends to zero, instead, we recover the case of fixed metabolic strategies and thus the CEP will determine the fate of the community. As shown in Fig 5, the distribution of stationary species’ populations can indeed change sensibly with the adaptation velocity *d*. On the other hand, if the system is subject to variable environmental conditions like the ones discussed previously (i.e. $\vec{s}\left(t\right)$ changes with time), as *d* increases the species’ are more able to promptly respond to perturbation and thus their populations will be less variable (see Figure K in S1 Text).

## Discussion

Community dynamics in the presence of multiple resources has traditionally been modeled via the MacArthur’s consumer-resource model, which prescribes the temporal dynamics of the population abundances of different competing species and of the resource densities. At present, the rates at which different species uptake different resources in such models have been treated as fixed parameters, in contrast with the experimental evidence that microbes can dynamically adapt nutrient uptake rates in response to environmental conditions. With fixed metabolic strategies, we find that the MacArthur’s consumer-resource model cannot reproduce the growth dynamics of a single microbial species in the presence of two resources, which shows that one must account for the temporal dynamics of nutrient uptake even in very simple ecological settings. For more complex communities, the MacArthur’s model with fixed metabolic strategies reproduces the so-called Competitive Exclusion Principle, whereby only *m* ≤ *p* species can survive if *p* resources are present, an observation that is in contrast with abundant empirical observations.

To understand if and how the classical MacArthur’s consumer-resource model can describe the coexistence of multiple competing species, we have introduced dynamic metabolic adaptation such that each species varies its metabolic strategies to maximize its growth rate. This new theoretical framework provides therefore a unified description of dynamic metabolic adaptation and community-level coexistence. We show that consumer-resource models with adaptive metabolic strategies can quantitatively describe the growth of a single microbial species on multiple resources through a fit of experimental data that gives physiologically reasonable best fit estimates for the model parameters. Furthermore, we show that the adaptive dynamics of metabolic strategies has a fundamental impact on species coexistence: the coexistence time interval of multiple species competing for few resources diverges as the characteristic timescale ratio (CTR) becomes less and less variable across different species. We suggest that this requirement is compatible with the experimental measurements of the scaling of vital and metabolic rates with body size that led to the metabolic theory of ecology [51], according to which the rates involved in the CTR depend only on the characteristic mass of the species. Fluctuations around such scaling patterns can be non-negligible [52–54] and thus it is likely that the coefficient of variation, Σ/〈*Q*〉, in natural communities is not exactly equal to zero. Thus, it is reasonable to postulate that such patterns may be responsible for reducing the coefficient of variation and thus prolonging extinction times, giving time to other processes not explicitly considered here to promote species coexistence, such as trade-offs in life-history traits [55–57] (e.g. the fact that some species grow faster when resources are abundant and others when resources are scarce, or that some species grow fast on a primary resource while growing more slowly on a secondary one). Therefore, we suggest that dynamic metabolic adaptation and the metabolic theory of ecology provide a fundamental mechanism for promoting the coexistence of a large number of species on a limited number of resources. Without invoking the metabolic theory of ecology, each species would have its own CTR and extinctions would be unavoidable, ultimately leading to the CEP. We find that the crucial parameter controlling the rate at which biodiversity is lost is the adaptation velocity, such that the CEP holds when dynamic metabolic adaptation is too slow with respect to the population dynamics.

Although we have focused only on competitive interactions, these are clearly not the only kind of interactions found in natural communities. Recent studies have shown that phenomena such as cross-feeding and syntrophy are ubiquitous in the microbial world [58], and have crucial roles in shaping the structure and function of microbial communities [6, 31, 59]. Future work will therefore be dedicated to incorporating cross-feeding and other types of inter-specific interactions in our theoretical framework. It has also been recently found that natural microbial communities are often composed of metabolically distinct and interdependent groups of species, each specialized in a particular function [2, 4, 60–62]. While the properties of interconnected ecological networks have been investigated in the past [63], a possible future development of our work consists in investigating if a modular organization of microbial species according to their metabolic function can be an emergent property of microbial communities, and in which conditions it stabilizes and is beneficial for such systems.

## Methods

### Comparison between the model and experimental measures of diauxic growth curves for *S. cerevisiae*

The *S. cerevisiae* strain used in this study, yAG47, is identical to strain yJHK459 of [64] and is in the W303 background. Its genotype is *MATa*, *can1-100*, *ura3* Δ0, *BUD4-S288C*. A culture of yAG47 was grown overnight in complete synthetic medium (CSM) + 2% (w/v) glucose. 1 mL of the overnight culture was spun down and resuspended in CSM + 0.5% (w/v) galactose to a concentration of 1.6 ⋅ 10^5^ cell/mL. Eight wells of a 96-well plate were inoculated with 150 *μ*L of the resuspended culture and incubated with constant shaking at 30°C in a plate reader. The 96-well plate was sealed with a sealing membrane that allowed gas exchange. The temperature on the top of the 96-well plate was kept at 31°C to avoid condensation on the membrane. Optical density (OD) measurements were taken every 10 min, for a total duration of about 70 h. To build the calibration curve used to convert OD to cell density, 1.4 mL of the same overnight culture were spun down and resuspended in 1 mL of CSM + 0.5% (w/v) galactose. The density of this suspension was measured using a Coulter counter and serial dilutions of this suspension were inoculated in a 96-well plate covered with the same sealing membrane used for the growth curve measurement. The OD of the wells containing the serial dilution of the suspension was measured after equilibration to 30°C using the same plate reader used to measure the growth curves, and these measurements were used to build the calibration curve converting OD to cell density.

When *S. cerevisiae* is grown on galactose as the primary carbon source, the sugar is partially respired and partially fermented. Yeast cells excrete ethanol as a byproduct of fermentation, which can then be used as a carbon resource. For this reason, in order to describe such system we use our adaptive consumer-resource model with *m* = 1, *p* = 2 and we slightly modify the equations for the temporal dynamics of ethanol concentration to take into account the fact that this resource is not initially present in the system but is produced by the yeast. In particular, the equations we used in order to describe the system are (5–9), where in (7) we have inserted an ethanol production rate that is proportional to the galactose consumption rate; in other words, *Y* can be interpreted as the galactose-to-ethanol yield.

$$\begin{array}{c} \dot{n}=n(v_{\text{gal}}\alpha_{\text{gal}}\frac{c_{\text{gal}}}{K_{\text{gal}}+c_{\text{gal}}}+v_{\text{eth}}\alpha_{\text{eth}}\frac{c_{\text{eth}}}{K_{\text{eth}}+c_{\text{eth}}}-\delta )\;\;, \end{array}$$ (5)

$$\begin{array}{c} {\dot{c}}_{\text{gal}}=-n\alpha_{\text{gal}}\frac{c_{\text{gal}}}{K_{\text{gal}}+c_{\text{gal}}}\;\;, \end{array}$$ (6)

$$\begin{array}{c} {\dot{c}}_{\text{eth}}=-n\alpha_{\text{eth}}\frac{c_{\text{eth}}}{K_{\text{eth}}+c_{\text{eth}}}+Y\cdot n\alpha_{\text{gal}}\frac{c_{\text{gal}}}{K_{\text{gal}}+c_{\text{gal}}}\;\;, \end{array}$$ (7)

$$\begin{array}{c} {\dot{\alpha }}_{i}=\alpha_{i}d\delta [v_{i}\frac{c_{i}}{K_{i}+c_{i}}-\Theta (\frac{\alpha_{\text{gal}}+\alpha_{\text{eth}}}{Q\delta }-1)\frac{1}{\alpha_{\text{gal}}+\alpha_{\text{eth}}}\cdot \\ \;\cdot (v_{\text{gal}}\alpha_{\text{gal}}\frac{c_{\text{gal}}}{K_{\text{gal}}+c_{\text{gal}}}+v_{\text{eth}}\alpha_{\text{eth}}\frac{c_{\text{eth}}}{K_{\text{eth}}+c_{\text{eth}}})]\;i=\text{gal,}\;\text{eth} \end{array}$$ (8)

$$\begin{array}{c} \frac{\alpha_{\text{gal}}+\alpha_{\text{eth}}}{Q\delta }\leq 1\;\;, \end{array}$$ (9)

Notice that since the model has several parameters (10 in total, some of which are phenomenological) there can be several different choices that lead to apparently equivalent fits. We therefore used a Markov Chain Monte Carlo algorithm [39] to fit this consumer-resource model to the experimental measurements of the population density of *S. cerevisiae*, both in the case of adaptive and fixed metabolic strategies, and to estimate the posterior distributions of the parameters. The comparison between the data and the best fits in the two cases are shown in Fig 1, while the values of the parameters obtained are shown in Table A in S1 Text. We used the same algorithm to fit the model with fixed metabolic strategies. Even if the model with fixed metabolic strategies is technically capable of reproducing the data (at the cost of returning parameters with biologically unrealistic values, see Figure A and Table A in S1 Text), we can use the Akaike Information Criterion (AIC) [65] to compare how it performs against the model with adaptive metabolic strategies. In fact, if we call Δ_AIC_ ≔ AIC_adaptive_ − AIC_fixed_ the difference between the AIC in the two cases, it is possible to show [65] that exp(Δ_AIC_/2) is the relative likelihood of the two models, and as such measures the probability that the model with fixed metabolic strategies minimizes the information loss (i.e. it is a better fit to the data than the one with adaptive metabolic strategies). In our case, by comparing the fits shown in Fig 1A and Figure A in S1 Text we found Δ_AIC_ = −938, so the probability that the results could be better explained using the model with fixed metabolic strategies is infinitesimal, even if the curve can nevertheless reproduce a diauxic behavior. The situation is of course even more extreme if we compare the fits shown in Fig 1A and 1B, since in this case we find Δ_AIC_ = −1327. We show in Figure C in S1 Text the predicted temporal dynamics of the resources concentrations, metabolic strategies and also of the constraint (9) using the best fit parameters of the model with adaptive metabolic strategies.

## Supporting information

S1 Text Additional details and computations on the model. (PDF)

S1 Data Data on the growth of *S. cerevisiae* on galactose. (XLS)
