# Correction: Dynamic metabolic adaptation can promote species coexistence in competitive microbial communities

**DOI:** 10.1371/journal.pcbi.1008721

**Published:** 2021-02-10

**Authors:** The PLOS Computational Biology Staff

In the Supporting Information file S1 Text some references are not displayed correctly; instead of the appropriate references, the file contains question marks "?" A corrected S1 Text file with the correct references shown is available below. The publisher apologizes for the error.

## Supporting information

S1 Text Additional details and computations on the model. (PDF)

## References

[1] Pacciani-Mori L, Giometto A, Suweis S, Maritan A (2020) Dynamic metabolic adaptation can promote species coexistence in competitive microbial communities. PLoS Comput Biol 16(5): e1007896 10.1371/journal.pcbi.1007896 32379752 PMC7244184

